# Interpretable multimodal machine learning for diagnosis of drug-resistant tuberculosis

**DOI:** 10.3389/fdgth.2026.1828522

**Published:** 2026-07-30

**Authors:** Joan Jonathan Mnyambo, Amir Aly, Emmanuel Ifeachor, Yinghui Wei, Shang-Ming Zhou, Stephen Mullin

**Affiliations:** 1 School of Engineering, Computing and Mathematics, University of Plymouth, Plymouth, United Kingdom; 2 Department of Informatics and Information Technology, Sokoine University of Agriculture, Morogoro, Tanzania; 3 University of Exeter Medical School, Faculty of Health and Life Sciences, University of Exeter, Exeter, United Kingdom; 4 School of Nursing and Midwifery, University of Plymouth, Plymouth, United Kingdom; 5 Peninsula Medical School, University of Plymouth, Plymouth, United Kingdom

**Keywords:** chest x-ray, data-efficient image transformer (DEiT), drug-resistant tuberculosis, interpretability, shapley additive explanations (SHAP)

## Abstract

**Introduction:**

Early detection of drug-resistant tuberculosis (DR-TB) is critical for timely treatment and limiting transmission. Differentiating DR-TB from drug-sensitive TB (DS-TB) remains challenging due to overlapping manifestations and subtle differences in chest X-rays (CXRs).

**Methods:**

We developed an explainable multimodal machine learning framework integrating CXR embeddings from a Data-Efficient Image Transformer (DEiT) with patient-level data, including clinical, interpreted radiological, demographic, socioeconomic, and behavioral variables. Using 6,279 subjects from the TB Portals program, model performance was evaluated across three settings comprising CXR embeddings only, patient-level features only, and multimodal. Interpretability was analyzed using Shapley Additive Explanations (SHAP).

**Results:**

A multimodal XGBoost model achieved a precision-recall area under the curve (PR-AUC) of 0.9258 and receiver operating characteristic area under the curve (ROC-AUC) of 0.8718. CXR-embeddings-only classifiers performed lower (PR-AUC: 0.8334; ROC-AUC: 0.7369), while patient-level XGBoost yielded highest performance. The multimodal model improved interpretability through complementary structural information. SHAP identified CXR embeddings as primary global drivers. Among patient-level variables, daily contacts and employment status were the strongest contributors, reflecting exposure intensity and socioeconomic conditions. Comorbidity showed moderate effects, while education and case definition had smaller contributions. Key radiological factors included non-tuberculosis abnormalities and mediastinal lymph node involvement, whereas medium-density stabalized fibrotic nodules, medium nodules, and small cavities demonstrated minimal association. Interaction analysis revealed higher risk with increasing comorbidity in the presence of morphological alterations; BMI and age exhibited modest influence. Independent review by two clinicians confirmed plausibility and consistency of SHAP explanations.

**Discussion:**

This interpretable multimodal framework supports early DR-TB detection and targeted interventions in high-burden settings.

## 1 Introduction

Rapid and accurate detection of drug-resistant tuberculosis (DR-TB) remains a major global health challenge. Early distinction between DR-TB and drug-sensitive tuberculosis (DS-TB) is essential to guide appropriate therapy, reduce ongoing transmission, and improve patient outcomes (1). In 2024, around 390,000 people developed multidrug-resistant or rifampicin-resistant tuberculosis (MDR/RR-TB) worldwide, with a marked disparity between patient groups, approximately 16% of previously treated cases were MDR/RR-TB relative to 3.2% among new diagnoses (2).

Current diagnostic approaches, including molecular assays and culture-based drug susceptibility testing, may be limited in low-resource settings by cost, infrastructure requirements, and delayed turnaround times (3, 4). Delayed identification of resistance can result in ineffective treatment, prolonged infectiousness, and increased morbidity (1). These constraints highlight the importance of complementary strategies that support earlier prediction of drug resistance and assist clinical decision-making.

Artificial intelligence (AI), particularly machine learning (ML), provides a promising approach to the prediction of resistant tuberculosis by identifying complex patterns in diverse datasets that traditional methods may overlook (5, 6). However, most existing models rely on a single data type and lack interpretability, limiting clinician confidence and practical applicability (7, 8). Robust, multimodal approaches with transparent reasoning are therefore needed to improve the accuracy, reliability, and clinical utility of DR-TB diagnosis.

This study presents an interpretable multimodal model that integrates CXR embeddings extracted using a Data-Efficient Image Transformer (DEiT) with patient-level features, including clinical, radiologically interpreted, demographic, socioeconomic, and behavioral variables. Compared with conventional convolutional neural networks (CNNs), which primarily capture local spatial features, transformer-based architectures enable more effective modeling of long-range dependencies in chest radiographs, where pathological patterns such as cavitation and nodules may be spatially dispersed (9). Among transformer-based approaches, standard Vision Transformers (ViTs), like CNNs, are typically data-intensive and prone to overfitting in small medical imaging datasets (10), while hybrid CNN–Transformer architectures introduce additional architectural complexity and computational overhead without consistently demonstrating superior performance in low-data regimes (11). In contrast, DEiT improves data efficiency through a knowledge distillation strategy from a convolutional teacher network, allowing it to retain the representational strengths of transformers while maintaining robustness in limited-data settings typical of resource-constrained environments (12).

DEiT captures subtle spatial patterns, such as cavitation and nodules, that conventional convolutional neural networks (CNNs) may miss, and performs effectively even on small datasets typical of resource-limited settings (9). The dataset was obtained from the TB Portals program, an international repository of tuberculosis cases (13). CXR embeddings are integrated with structured patient-level features within a transparent machine learning framework to enhance diagnostic accuracy. Model interpretability is ensured using SHAP, which provides feature- and modality-specific explanations to support evidence-based diagnostic decision-making (14–16).

In this work, the prediction task is formulated as a binary classification problem distinguishing DR-TB from DS-TB. This framing is motivated by the clinical objective of early identification of drug resistance among confirmed tuberculosis patients, where treatment decisions differ substantially between resistant and sensitive cases. Consequently, the dataset used in this research includes only microbiologically or clinically confirmed TB cases, while healthy individuals and other pulmonary diseases are excluded.

The exclusion of non-TB and alternative respiratory conditions reflects the structure of the TB Portals dataset, which is primarily designed for tuberculosis-specific research cohorts rather than general population screening. While this setting enables focused modeling of resistance-related patterns, it also implies that the proposed framework is not intended for TB diagnosis or differential diagnosis against other lung diseases, but rather for resistance classification within confirmed TB patients.

Major contributions of this work include:

- Interpretable multimodal prediction. Integrating CXR embeddings with patient-level features overcomes the limitations of single-datatype models, enhances DR-TB classification, and provides transparent, actionable insights.
- Feature impact analysis. Leveraging SHAP ranking across CXR embeddings, clinical, interpreted radiological, demographic, socioeconomic, and behavioral variables offers an explainable view of the most influential predictors of DR-TB outcomes.
- Trustworthy AI framework. Combining CXR embeddings and patient-level features in a clinically meaningful predictive model supports reliable decision-making and facilitates potential real-world adoption in DR-TB care.

By using multi-source health data within an interpretable machine learning framework, this study addresses challenges in DR-TB detection and supports more personalized care. The approach is designed to be scalable and applicable in heterogeneous, resource-limited settings.

This paper is structured as follows: Section 2 reviews prior research on drug-resistant tuberculosis prediction and interpretable machine learning, Section 3 describes the methodological framework; Section 4 presents results and analysis; Section 5 discusses the main findings; and Section 6 concludes with suggestions for future research.

## 2 Related studies

Machine learning approaches for drug-resistant tuberculosis diagnosis have leveraged radiological, clinical, and demographic data to identify key predictive factors, including the extent of lung involvement, contact history, and disease type. Early studies employing support vector machines (SVM) and random forest classifiers reported predictive accuracies of 72.3% and 75.0%, respectively (17, 18). Although these methods demonstrated moderate predictive capability, their limited interpretability restricted insight into feature-level decision mechanisms, thereby reducing clinical trust and limiting translational applicability (6, 19).

More recent studies have extended DR-TB modeling through multimodal learning frameworks that integrate clinical, imaging, and genomic information. These approaches have reported AUC values between 0.803 and 0.899, with classification accuracies reaching up to 92.4% (15, 20, 21). In parallel, advances in tuberculosis-focused deep learning have introduced transformer-based architectures and multimodal fusion strategies that improve representation learning across heterogeneous data sources. For instance, hybrid Vision Transformer (ViT) and convolutional neural network (CNN) models have demonstrated enhanced capability in multi-label tuberculosis abnormality detection from chest x-rays (22). Similarly, ViT-based frameworks augmented with post-hoc explainability methods such as Grad-CAM have achieved improved diagnostic performance while enabling localized visual interpretation of salient regions (23). Furthermore, multimodal foundation models such as PaliGemma-CXR integrate visual and clinical reasoning within a unified architecture, achieving strong diagnostic performance and improved generalization across tasks (24).

Although these methods primarily target general tuberculosis detection rather than DR-TB classification, their underlying architectural principles and multimodal representation learning capabilities are directly transferable to resistance-aware modeling. In particular, their ability to encode complex imaging phenotypes and integrate heterogeneous clinical signals provides a strong foundation for extending these approaches to DR-TB prediction.

Concurrently, imaging-based machine learning has progressed through radiomics and deep learning approaches that extract quantitative representations from chest x-rays (CXR) and computed tomography (CT) scans (25–28). Contemporary convolutional neural networks and transformer-based models further enhance performance by capturing hierarchical and global contextual features associated with tuberculosis pathology. Nevertheless, these approaches remain limited by insufficient interpretability and a lack of integration with patient-level clinical and genomic information, particularly in DR-TB characterization tasks.

To address the growing need for transparency in high-performing predictive systems, explainable AI techniques have been increasingly adopted. SHAP-based analyses applied to genomic datasets demonstrate that interpretable predictions can be achieved even in high-dimensional single-modality settings. Using single nucleotide polymorphism (SNP) features derived from whole-genome sequencing, models such as XGBoost and artificial neural networks achieved sensitivities of 77%–95% and specificities of 94%–99%, respectively, while gradient boosting classifiers reached performance levels up to 97%. Of note, SHAP enabled identification of mutation-level contributions driving resistance predictions (29, 30). These findings highlight the potential of SHAP to reconcile predictive accuracy with interpretability in DR-TB modeling.

However, existing SHAP-based approaches remain largely restricted to single-source data and do not adequately capture cross-modal interactions among clinical, imaging, and genomic variables. Consequently, even high-performing models may fail to represent the full complexity of DR-TB progression, thereby limiting clinical interpretability and adoption in real-world settings (6, 8).

Overall, the literature reveals a consistent trade-off: classical machine learning approaches offer interpretability at the expense of predictive performance, whereas deep learning and multimodal architectures achieve superior accuracy but remain largely opaque. Existing explainability techniques partially mitigate this limitation but are predominantly applied within single-modality frameworks and fail to model cross-domain feature interactions.

Motivated by these limitations, the present study develops a multimodal framework that integrates chest x-ray embeddings with structured patient-level features. SHAP-based attribution is employed to provide both feature-level and modality-level interpretability across imaging, clinical, radiological, demographic, socioeconomic, and behavioral domains. This design aims to jointly optimize predictive performance and interpretability, thereby enabling clinically reliable and decision-supportive DR-TB diagnosis.

This study should therefore be interpreted as a focused DR-TB classification framework rather than a full clinical screening system. In real-world deployment, models would need to operate in a broader diagnostic setting that includes healthy individuals and patients with other pulmonary conditions such as pneumonia, lung cancer, or non-tuberculous infections. The exclusion of such cases may limit external validity and positions the current work as a controlled, proof-of-concept study on resistance differentiation within confirmed TB populations.

However, the proposed framework remains clinically relevant as DR-TB determination is typically performed after TB suspicion or diagnosis. Nevertheless, future work should extend the model to multi-class or hierarchical classification settings that include non-TB and other pulmonary diseases, enabling robust triage-level deployment.

From a generalization perspective, incorporating heterogeneous non-TB cases during training would likely improve model calibration and reduce false positive rates in real-world settings. Domain adaptation or incremental learning strategies could further enhance robustness when transitioning from curated datasets to clinical screening environments.

## 3 Methods

**Figure 1:**
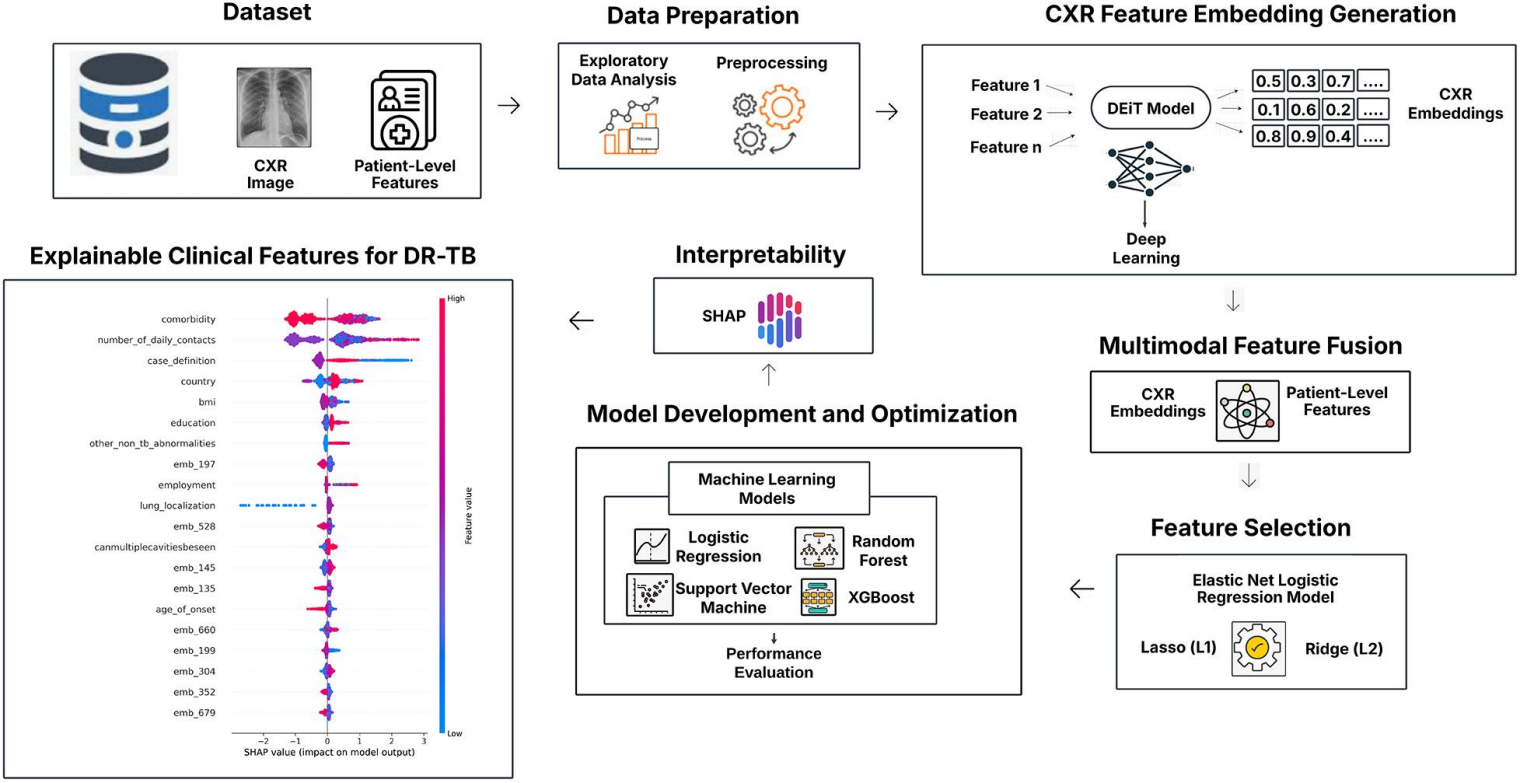
Interpretable multimodal machine learning workflow for DR-TB prediction.

An interpretable multimodal machine learning model was developed to predict drug-resistant tuberculosis by integrating chest x-ray embeddings extracted using a Data-Efficient Image Transformer with patient-level features. The DEiT model, pretrained on ImageNet and fine-tuned on TB Portals CXR images, generates high-dimensional embeddings capable of discriminating drug-resistant from drug-sensitive tuberculosis. These embeddings were fused with patient-level variables, including clinical, interpreted radiological, demographic, socioeconomic, and behavioral data, to construct a comprehensive multimodal dataset for predictive modeling. Model interpretability was achieved using SHAP to quantify feature contributions and identify the most influential predictors, complemented by permutation feature importance for global validation and Local Interpretable Model-agnostic Explanations (LIME) for instance-level interpretation. The overall workflow is illustrated in Figure 1.

### 3.1 Data description and preprocessing

Chest x-ray images and patient records were obtained from the TB Portals program, covering 11 high-TB-prevalence countries: India, South Africa, Georgia, Ukraine, Moldova, Belarus, Kazakhstan, Romania, Kyrgyzstan, Nigeria, and Azerbaijan (13). Categorical variables were encoded for modelling, while images were preprocessed using a standardized pipeline to ensure uniformity.

Interpreted radiological features were derived from the TB Portals dataset, which provides detailed chest x-ray annotations generated by radiologists. These annotations follow standard radiological conventions, with the patient’s right lung displayed on the left side of the image and vice versa. The provided features capture clinically meaningful patterns, including cavitation, infiltrates, nodules, and other lung abnormalities, and were incorporated directly into the multimodal dataset for modeling (13).

Table 1 presents TB cases by gender, age, and resistance type. The dataset comprised 6,279 patients, of whom 4,091 (65.2%) were DR-TB and 2,188 (34.8%) DS-TB, yielding an imbalance ratio of 1.87. The cohort was predominantly male (4,662, 74.2%), with females representing 1,617 (25.8%). Stratified by resistance type, DR-TB included 3,053 males and 1,038 females, whereas DS-TB comprised 1,609 males and 579 females.

**Table 1:** Demographic and resistance profiles of TB patients by gender, age (years), and resistance status.

Features	Groups	Total	Female	Male
		6,279	1,617 (25.8%)	4,662 (74.2%)
Age (years)	Min	1	1	1
	Max	93	93	93
	Mean ± SD	43.3 ± 14.0	40.8 ± 16.5	44.2 ± 13.0
Type of resistance	DR-TB	4,091 (65.2%)	1,038 (64.2%)	3,053 (65.5%)
	DS-TB	2,188 (34.8%)	579 (35.8%)	1,609 (34.5%)

Age of onset ranged from 1 to 93 years. Overall, the mean ± SD was 43.3 ± 14.0 years, with a median (IQR) of 42.0 (32–55) years. By gender, males had a mean ± SD of 44.2 ± 13.0 years (median 43, IQR 35–53) and females 40.8 ± 16.5 years (median 37, IQR 29–49), showing broadly similar distributions.

Missing data were evaluated separately for numerical and categorical variables. Numerical entries such as BMI were missing in 28.99% of cases and were imputed using column means. Among categorical variables, missingness was highest for comorbidity (48.29%) and number of daily contacts (38.09%), and these entries were encoded as “Unknown,” under the assumption of Missing Completely at Random (MCAR) (31). Other variables had low levels of missingness, generally below 2.3%. This approach preserved cohort representativeness while minimizing potential bias. Chest x-ray images and clinical features were merged using unique patient identifiers, with categorical variables label-encoded and continuous variables standardized, producing a harmonized dataset suitable for downstream analyses.

**Figure 2:**
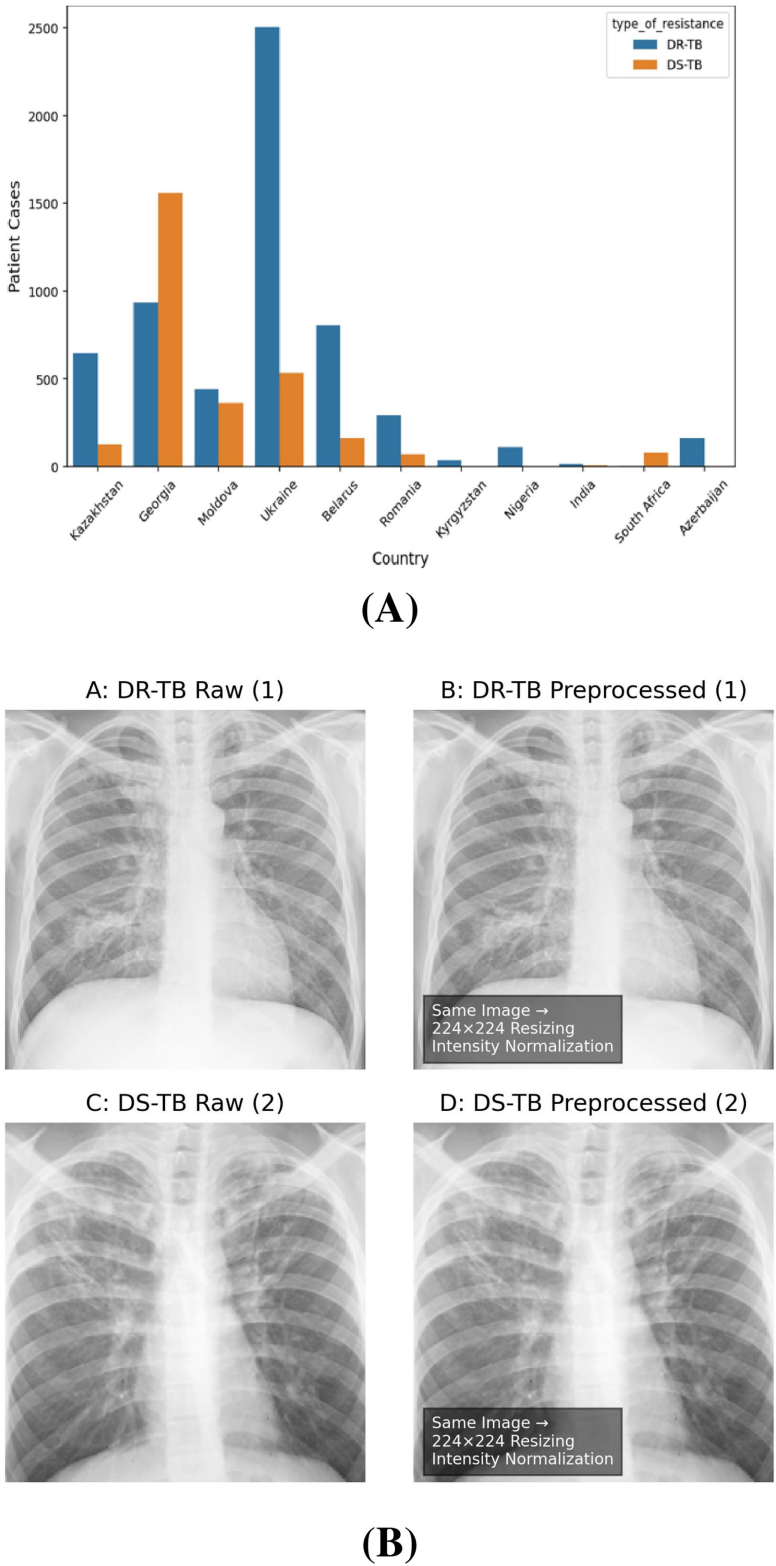
Overview of resistance distribution and chest x-ray samples. **(A)** Distribution of resistance types by country. **(B)** Raw and preprocessed chest x-ray images from DR-TB and DS-TB cases.

Figure 2A shows the distribution of TB resistance types across countries, highlighting substantial regional variation. DR-TB predominates in Eastern Europe (Ukraine, Georgia, Belarus, Moldova, Romania) and Central Asia (Kazakhstan, Azerbaijan, Kyrgyzstan), while drug-sensitive cases are most common in South Africa. Some countries, including India, parts of Central Asia, and Nigeria, contained only DR-TB cases. To mitigate potential biases in model development, sensitivity analyses were conducted by either removing the country variable or excluding countries with only a single resistance type. Representative chest x-ray samples from drug-resistant and drug-sensitive cases are shown in Figure 2B, illustrating the imaging data used for model development alongside the preprocessing steps applied prior to analysis. Demographic distributions, including age and gender, were examined to ensure transparency regarding cohort composition. Presenting both geographic and demographic details allows readers to assess potential imbalances and interpret model performance in context.

For modeling, a single baseline CXR per patient (the first available scan) was selected. This approach ensures that the image represents the patient’s initial visit when tuberculosis infection was confirmed, marking the start of treatment, while maintaining consistency across patients and avoiding bias from multiple scans per individual (32). Across the cohort, a total of 8,844 CXRs were available, with most patients having more than one scan.

No explicit lung segmentation or image-level artifact removal was performed; instead, a standardized preprocessing pipeline was applied to all images to ensure uniformity. This included aspect-ratio-preserving resizing, center cropping to 224×224 pixels, conversion to RGB format, and ImageNet-based intensity normalization. These steps reduce variability in image scale and intensity while preserving the full radiographic context. Images were subsequently used as input for feature extraction using DEiT. Associations between patient characteristics and resistance status were assessed using Levene’s test for equal variances, Welch’s t-test for continuous data, and chi-square tests for categorical measures.

### 3.2 Deep learning-based extraction of chest x-ray feature embeddings

High-level CXR embeddings were extracted from the chest x-rays described in Section 3.1 using a DEiT-B/16 model pretrained on ImageNet-1k and fine-tuned to differentiate DR-TB from DS-TB. The DEiT-B/16 architecture consists of 12 transformer encoder layers, with 12 attention heads and an embedding dimension of 768.

Each CXR was divided into 196 non-overlapping 16×16 patches arranged in a 14×14 grid, preserving spatial information while maintaining computational efficiency. These patches were flattened and linearly projected into a d-dimensional embedding space:

$$x_{p}=\text{Linear}(\text{Flatten}(P_{i})),\;\text{\textbackslash{},for }i=1,2,\ldots ,196$$ (1)

Equation 1 defines the mapping of each flattened patch into a fixed-dimensional embedding vector used as input to the DEiT encoder. Here, $x_{p}$ represents the embedding of the i-th patch. A learnable class (CLS) token was prepended to the sequence of embeddings, and positional encodings were added to retain spatial relationships. The resulting sequence was processed through multiple transformer encoder layers.

Multi-head self-attention was applied to the embeddings using Equation 2:

$$\text{Attention}(Q,K,V)=\text{softmax}\left(\frac{QK^{⊤}}{\sqrt{d_{k}}}\right)V$$ (2)

In this formulation, the embeddings are used to construct the query (Q), key (K), and value (V) matrices for the attention mechanism.

The pretrained DEiT backbone was frozen, and only the classification head was fine-tuned on the target dataset. The original head was replaced with a custom multi-layer perceptron (MLP) consisting of a fully connected layer reducing the 768-dimensional CLS token to 64 units, followed by a ReLU (Rectified Linear Unit) activation, dropout, and a final linear layer producing a single output for binary classification.

**Figure 3:**
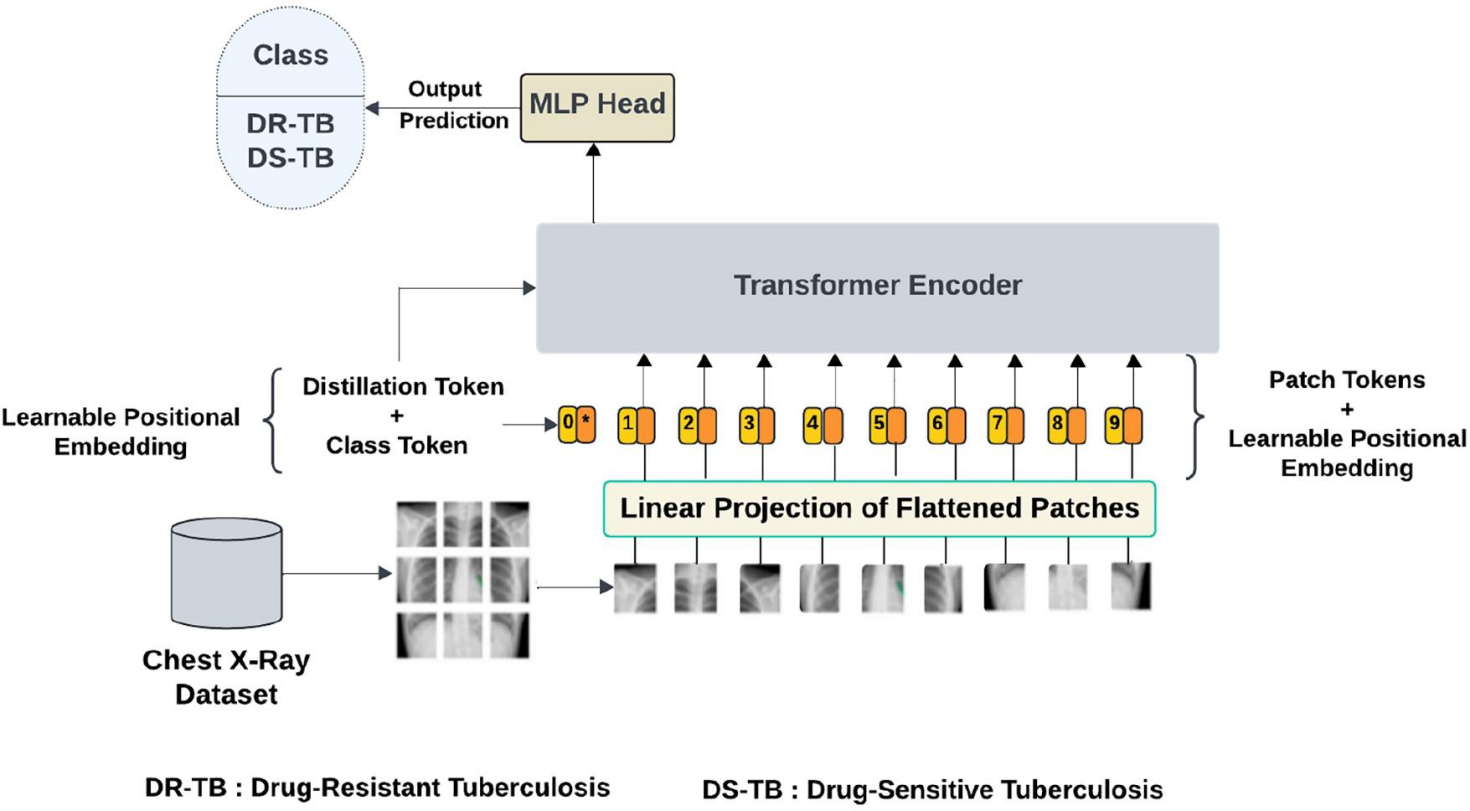
Schematic of the DEiT model used for chest x-ray feature extraction. Reproduced from “DEiT model architecture for detecting drug-resistant tuberculosis using chest X-rays” by Joan Jonathan Mnyambo, Amir Aly, Shang-Ming Zhou, Yinghui Wei, Stephen Mullin and Emmanuel Ifeachor, licensed under CC BY-NC-ND 4.0.

A single 768-dimensional image-level embedding was obtained from the CLS token at the output of the final transformer encoder layer, prior to the classification stage, summarizing all patches. These embeddings were subsequently combined with patient-level features for multimodal modeling as illustrated in Figure 3.

### 3.3 Multimodal feature fusion

Following preprocessing and CXR feature extraction in Sections 3.1 and 3.2 complementary information from both modalities was integrated using a joint feature-level fusion strategy. This approach allows simultaneous modeling of CXR and patient-level features and improves predictive performance compared with strategies that process each modality independently (6).

For each patient i, the CXR features $z_{i}\in R^{d}$ and patient-level features $c_{i}\in R^{m}$ were concatenated to form a multimodal fused embedding:

$$f_{i}=[z_{i}\;|\;c_{i}]\in R^{d+m}$$ (3)

Equation 3 represents the linear projection of flattened image patches into the embedding space. The multimodal fused embedding $f_{i}$ is then fed into downstream predictive models, allowing the learning of joint representations that capture complementary patterns across both CXR and patient-level features.

where d=768 and m=54 denote the dimensionality of the CXR embeddings and patient-level features, respectively, and “|” indicates vector concatenation. The resulting multimodal dataset comprised 7,955 samples with a total of 822 features, including 768 CXR embeddings and 54 patient-level features.

By fusing features at the embedding level, the model can leverage complementary information across modalities, improving overal reliability and enabling more interpretable predictions (33).

### 3.4 Feature selection

To minimize redundancy and improve interpretability, feature selection was performed on the high-dimensional multimodal features described in Section 3.3. The dataset was split into an 80% training set and a 20% independent test set using stratified sampling based on resistance status, preserving class balance and ensuring representativeness for model evaluation. Prior to feature selection, numeric features were standardized (mean-centered and scaled to unit variance) and categorical features were appropriately encoded for model input (34).

Using nested stratified 5-fold cross-validation on the training set, feature selection was implemented with an Elastic Net logistic regression model. By combining $L_{1}$ (LASSO, Least Absolute Shrinkage and Selection Operator) and $L_{2}$ (Ridge) penalties, the model promotes sparse selection and stabilizes coefficients when predictors are correlated (35–37).

Hyperparameters, including the regularization strength (λ) and the $L_{1}-L_{2}$ mixing parameter (α), were tuned using inner-fold grid search, optimizing the ROC-AUC, which is robust to class imbalance (9). The parameter α controls the balance between $L_{1}$ and $L_{2}$ regularization, where α=0 is pure $L_{2}$, α=1 is pure $L_{1}$, and intermediate values combine both penalties. The most frequently selected values across outer folds (λ=100, α=0.1) were retained to ensure feature stability (38).

Employing these consensus hyperparameters, the final Elastic Net model was fitted to the entire training-validation set. Features with absolute coefficients $|\beta |>1\times 10^{-4}$ were retained, removing near-zero contributions while preserving informative predictors. The threshold of 1e-4 was selected as a conservative cut-off within a stable performance region identified through sensitivity analysis. Across a wide range of thresholds (1e-6 to 1e-3), both model performance and feature composition remained highly consistent, indicating that this value provides a balanced trade-off between sparsity and stability. Elastic Net performs simultaneous shrinkage and variable selection, yielding sparse and interpretable solutions that are well suited for high-dimensional biomedical data (39, 40). For sample i, the predicted probability of drug resistance is given in Equation 4:

$${\hat{\;p}}_{i}=\frac{e^{{\hat{\beta }}_{0}+{\hat{\beta }}^{T}X_{i}}}{1+e^{{\hat{\beta }}_{0}+{\hat{\beta }}^{T}X_{i}}}$$ (4)

The coefficient vector $\hat{\beta }$ is estimated by minimizing the Elastic Net objective, as shown in Equation 5:

$$\hat{\beta }=\arg\min_{\beta }\left\{\begin{array}{cc} -\sum_{i=1}^{n}y_{i}\log({\hat{\;p}}_{i})-\sum_{i=1}^{n}(1-y_{i})\log(1-{\hat{\;p}}_{i})\\ & \;+\lambda \;\alpha \;\|\beta {\|}_{1}+\frac{\lambda }{2}(1-\alpha )\|\beta {\|}_{2}^{2} \end{array}\right\}$$ (5)

Here, $X\in R^{n\times p}$ denotes the feature matrix, $y\in \{0,1{\}}^{n}$ the binary resistance outcome, λ>0 the regularization strength, and α∈[0,1] the $L_{1}$-$L_{2}$ mixing parameter. The first component captures the logistic loss, while the second part applies Elastic Net regularization to encourage sparsity and maintain coefficient stability (40, 41).

Table 2 summarizes the patient-level features retained after Elastic Net feature selection, grouped by domain. Behavioral, clinical, demographic, socioeconomic, and interpreted radiological variables were preserved alongside selected CXR embeddings. This curated feature set balances predictive performance and interpretability and was used for downstream model development and independent test-set evaluation in Section 3.5.

**Table 2:** Feature groups, counts, and corresponding features in the dataset.

Feature group	Count	Features
Behavioral	1	number_of_daily_contacts
Clinical	3	case_definition, bmi, comorbidity
Demographic and Socioeconomic	5	age_of_onset, gender, country, education, employment
Interpreted radiological features		
Cavitation	4	totalcavernum, smallcavities, isanylargecavitybelongtoamultisextantcavity, canmultiplecavitiesbeseen
Infiltrate	6	affect_level, overall_percent_of_abnormal_volume, posttbresiduals, infiltrate_lowgroundglassdensity, infiltrate_mediumdensity, infiltrate_highdensity
Inflation/Collapse	1	lung_capacity_decrease
Nodule	7	mediumnodules, hugenodules, isanyclusterednoduleexists, aremultiplenoduleexists, lowgroundglassdensityactivefreshnodules, mediumdensitystabalizedfibroticnodules, nodicalcinatum
Other Lung Abnormality	8	lung_localization, shadow_pattern, other_non_tb_abnormalities, affect_pleura, pleural_effusion_percent_of_hemithorax_involved, ispleuraleffusionbilateral, are_mediastinal_lymphnodes_present, anomaly_of_mediastinum_vessels_develop

### 3.5 Model development, optimization, and evaluation

Features selected in Section 3.4 were used to train predictive models under three settings: CXR embeddings only, patient-level features only, and multimodal (CXR embeddings and patient-level features) inputs. The training set (80% of the data) was used for nested stratified 5-fold cross-validation, while the independent test set (20%) was reserved for final evaluation.

Four machine learning classifiers were evaluated: Logistic Regression (LR), Random Forest (RF), eXtreme Gradient Boosting (XGBoost), and Support Vector Machine (SVM). LR served as a linear baseline, while RF and XGBoost captured nonlinear feature interactions. SVM was included due to its strong performance in high-dimensional feature spaces (42, 43). This selection balances interpretability, nonlinear modeling capacity, and effectiveness in structured biomedical data.

Nested stratified 5-fold cross-validation was applied for all classifiers. For LR, RF, and SVM, hyperparameters such as regularization strength, number of trees, maximum depth, kernel type, and cost parameter were optimized. Class imbalance was addressed using class_weight=’balanced’. For XGBoost, learning rate, maximum depth, subsample ratio, and column sampling were tuned, with scale_pos_weight used to handle class imbalance and early stopping based on validation ROC-AUC to prevent overfitting (34). The DR-TB class was designated as the positive class. Decision thresholds were selected by minimizing the absolute difference between precision and sensitivity. Hyperparameter tuning was performed via grid search within the inner folds, while outer folds provided unbiased performance estimates.

Model performance was primarily assessed using PR-AUC to account for class imbalance, followed by ROC-AUC, balanced accuracy, F1-score, sensitivity, and specificity. 95% confidence intervals were estimated using 1,000 bootstrap iterations. Pairwise ROC-AUC comparisons were performed using the DeLong test.

#### 3.5.1 Deep learning models

To complement the feature-based machine learning models and provide a direct deep learning benchmark, four transfer learning-based imaging models were evaluated: DEiT-B/16, ViT-B/16, DenseNet-121, and ResNet-50.

Transformer-based architectures included DEiT-B/16 and ViT-B/16. DEiT-B/16 is a data-efficient Vision Transformer that learns global contextual representations from image patches using multi-head self-attention. It incorporates distillation and strong regularization, making it suitable for limited medical imaging data (9). In addition, ViT-B/16 was evaluated as a standard Vision Transformer baseline. Similar to DEiT, ViT-B/16 divides input images into fixed-size patches and models global relationships using self-attention, but without the data-efficient distillation strategy, making it more data-intensive and typically requiring larger training datasets for effective performance (44). This allows comparison between a conventional transformer and a data-efficient variant in the context of medical imaging.

$$\text{Attention}(Q,K,V)=\text{softmax}\left(\frac{QK^{⊤}}{\sqrt{d_{k}}}\right)V,$$ (6)

Equation 6 represents the scaled dot-product attention mechanism, where $d_{k}$ denotes key dimensionality.

CNN baselines consisted of DenseNet-121 and ResNet-50. DenseNet-121 was used due to its dense connectivity, which enhances feature reuse and gradient propagation (45, 46). In DenseNet architectures, each layer receives feature maps from all preceding layers as illustrated in Equation 7:

$$x_{l}=H_{l}([x_{0},x_{1},\ldots ,x_{l-1}]),$$ (7)

where $H_{l}(\cdot )$ represents a composite of batch normalization, ReLU, and convolution operations. ResNet-50 employs residual connections to mitigate vanishing gradients, enabling deeper network training, as expressed in Equation 8,

y=F(x)+x, (8)

where F(x) is the residual mapping.

All models were initialized with ImageNet pre-trained weights and adapted for binary classification using a single linear output neuron. Training followed a consistent protocol across models using binary cross-entropy with logits loss, AdamW optimization ($lr=3\times 10^{-4}$, weight decay $=10^{-2}$), cosine annealing over 50 epochs, and early stopping based on validation ROC-AUC (patience = 10). Only classification heads were trained, while backbone weights were frozen. Standard ImageNet normalization and data augmentation (random resized cropping, horizontal flipping, and color jittering) were applied.

More complex hybrid CNN–Transformer architectures and larger-scale variants were not considered in order to maintain a focused experimental design and ensure computational feasibility given the study setting and dataset size. These deep learning baselines enable comparison against classical machine learning and multimodal fusion approaches, assessing whether learned representations outperform structured feature-based models in DR-TB classification.

#### 3.5.2 Multimodal deep learning model

In addition to classical machine learning models, a multimodal deep learning architecture based on a multimodal multi-layer perceptron (MLP) was implemented to enable learned integration of heterogeneous data (47). Unlike early fusion approaches, which rely on direct concatenation of raw features, this model employs modality-specific encoders followed by a joint fusion network.

Equation 9 shows that each modality-specific encoder contains two fully connected layers that learn intermediate representations from the corresponding inputs. The CXR embeddings $z_{i}\in R^{d}$ and patient-level features $c_{i}\in R^{m}$ are processed separately:

$$h_{i}^{\left(z\right)}=f_{z}(z_{i}),\;h_{i}^{\left(c\right)}=f_{c}(c_{i}),$$ (9)

where $f_{z}(\cdot )$ and $f_{c}(\cdot )$ denote nonlinear transformations with ReLU activations.

The resulting modality-specific representations are concatenated into a joint feature vector:

$$h_{i}=[h_{i}^{\left(z\right)}\;|\;h_{i}^{\left(c\right)}],$$ (10)

which is passed through the fusion network to produce the final prediction:

$${\hat{y}}_{i}=g(h_{i}),$$ (11)

where g(⋅) is a fully connected network with a sigmoid output for binary classification. Equations 10 and 11 describe the fusion of modality-specific representations and the subsequent prediction stage.

The model was trained end-to-end using binary cross-entropy loss with class weighting to address class imbalance between DR-TB and DS-TB cases, and optimized using the Adam optimizer with early stopping based on validation loss. This architecture enables learning of complementary patterns across imaging and clinical modalities beyond simple feature concatenation. Of particular note, this multimodal MLP was included as a deep learning benchmark to enable direct comparison against classical machine learning fusion models.

### 3.6 Model interpretability

SHAP values were calculated for the final model using the TreeExplainer algorithm, optimized for ensemble tree-based models (48). Interpretability analyses were conducted on the independent test set to provide an unbiased assessment of feature contributions. For imaging-derived inputs, the DEiT embeddings represent high-dimensional latent feature representations learned from chest x-ray data. Individual embedding dimensions (e.g., emb_136, emb_736) do not correspond to explicit clinical or radiological concepts; rather, they encode abstract patterns learned by the network. Therefore, SHAP values applied to these features quantify their contribution to model predictions in latent space, but do not provide direct clinical interpretability of individual embedding dimensions.

For each sample i, the predicted probability $f(x_{i})$ is decomposed relative to the expected model output E[f(x)] as:

$$f(x_{i})-E[f(x)]=\sum_{\;j=1}^{M}\phi_{j}(i)$$ (12)

where $\phi_{j}(i)$ represents the Shapley value for feature j for sample i, indicating the feature’s individual contribution to the model’s prediction. Equation 12 formalizes the additive representation of the model output in terms of feature-wise contributions.

Global interpretability was assessed using SHAP on the full multimodal feature set. Summary (beeswarm) plots ranked features by mean absolute contribution and visualized effect distributions across samples from the independent test set (49). The top predictors were then examined by domain, with interpreted radiological features analyzed separately and behavioral, clinical, demographic, and socioeconomic features analyzed jointly. SHAP dependence plots explored nonlinear effects and interactions among key predictors, while correlation heatmaps examined linear relationships among selected radiological variables (50).

Local interpretability was evaluated using individual-level SHAP waterfall plots, which decompose single predictions into additive feature contributions and highlight the dominant drivers of specific model decisions (48). Test samples were ranked by predicted probability to identify high-risk cases for detailed inspection. Together, these analyses provide complementary global and patient-specific insights into the factors driving drug-resistant tuberculosis classification.

Permutation feature importance was additionally used as a model-agnostic validation of global feature relevance. This method estimates feature importance by measuring the reduction in ROC-AUC after randomly permuting each feature while keeping all others fixed, providing an independent robustness check of SHAP-based rankings (51).

LIME were applied as a complementary method for instance-level interpretability. LIME approximates the local decision boundary using a sparse linear surrogate model fitted on perturbed samples around each prediction, enabling comparison with SHAP-based local explanations and assessing the consistency of feature attributions at the individual level (52).

### 3.7 Clinical validation of SHAP-based model interpretations

To evaluate the plausibility of model explanations, a structured clinical review was conducted by two independent clinicians with expertise in tuberculosis and infectious diseases. Representative cases using patient-level data were inspected for consistency with established epidemiological, radiological, and pathological patterns of tuberculosis. Additionally, a case-based assessment of 10 representative samples was performed to further examine the interpretability and reliability of the model outputs.

## 4 Results

This section summarizes the outcomes of the modeling framework, including dataset characteristics, feature selection results, model performance, and interpretability analyses. Model performance is reported for CXR embeddings only, patient-level features only, and multimodal (CXR embeddings and patient-level features) inputs, allowing a direct comparison of predictive accuracy and discrimination on the independent test set. Findings highlight the benefits of combining CXR embeddings and patient-level features and conclude with an assessment of the most influential predictors as identified by SHAP values.

### 4.1 Patient demographic characteristics

Table 1, presented in Section 3.1 summarizes the study population, showing a predominantly male cohort, an adult age distribution, and a high proportion of drug-resistant tuberculosis. Associations between patient characteristics and resistance type are reported in Table 3. Levene’s test indicated unequal variances for age, prompting the use of Welch’s t-test, which revealed a significant age difference between DS-TB and DR-TB patients. Chi-square analysis identified a significant relationship between gender and resistance status.

**Table 3:** Statistical test outcomes for associations between patient characteristics and resistance type.

Feature	Measure/group	Test	p-value	Interpretation
Age (years)	Variance check	Levene’s test	$1.19\times 10^{-47}$	Unequal variances between resistance type
Age (years)	DS-TB vs. DR-TB	Welch’s t-test	$4.03\times 10^{-13}$	Age differs significantly between resistance type
Gender	DS-TB vs. DR-TB	Chi-square	$4.00\times 10^{-4}$	Significant association with resistance type

These univariate tests indicate that age and gender are associated with resistance type but do not capture complex, nonlinear interactions among CXR embeddings and patient-level features. Accurate prediction of DR-TB therefore requires integration of multiple data modalities. The multimodal model described in Sections 3.4, 3.5, 3.6 combines CXR embeddings , clinical, interpreted radiological, demographic, socioeconomic, and behavioral data, resulting in enhanced predictive performance and improved interpretability.

### 4.2 Feature selection outcomes

Elastic Net feature selection reduced the multimodal feature set from 822 to 413 variables, retaining features with coefficients above a threshold ($|\beta |>1\times 10^{-4}$), as described in Section 3.4 and summarized in Table 4. This step removed redundant and weakly contributing variables, improving model interpretability and reducing overfitting risk. The resulting feature space preserved both CXR embedding and patient-level predictors, achieving a PR-AUC of 0.8757 and ROC-AUC of 0.8052 prior to downstream classifier training. Performance remained stable across sensitivity thresholds, with only minimal variation in both PR-AUC and ROC-AUC despite small changes in feature count (413 to 409).

**Table 4:** Sensitivity analysis of feature selection thresholds and their effect on model performance.

Performance			Feature breakdown		
Threshold	PR-AUC	ROC-AUC	Total features	CXR embeddings	Patient-level
$1\times 10^{-6}$	0.8757	0.8052	413	378	35
$1\times 10^{-5}$	0.8757	0.8052	413	378	35
$1\times 10^{-4}$	0.8757	0.8052	413	378	35
$1\times 10^{-3}$	0.8719	0.8015	409	374	35

To further interpret the modality-wise effect of Elastic Net selection, we examined the proportion of selected features across modalities. The selected feature subset included a larger number of CXR embedding dimensions (378/768, 49.2%) compared to patient-level variables (35/54, 64.8%). This difference is expected given the substantially higher dimensionality of the embedding space relative to structured clinical data. While the absolute number of selected embedding features is higher, the relative selection rates differ across modalities; however, this pattern is driven by structural differences in the feature spaces rather than systematic modality preference, reflecting higher correlation within learned imaging-derived embeddings.

Predictive performance remained stable across feature selection thresholds, suggesting that Elastic Net induces a compression–informativeness trade-off by reducing embedding dimensionality while prioritising features most relevant for prediction. Elastic Net-based selection yields a compact feature set with preserved discriminative performance.

### 4.3 Comparative performance of classifiers

Performance differed across CXR embeddings only, patient-level features only, and multimodal (CXR embeddings and patient-level features) inputs, reflecting the influence of data modality and algorithm choice on DR-TB prediction as reported in Table 5. Across all CXR embedding-based models, performance was consistently moderate, with no classifier demonstrating a clear advantage. Both PR-AUC and ROC-AUC remained lower than those obtained using patient-level features, indicating limited discriminative information in imaging representations alone.

**Table 5:** Performance metrics for classical ML and multimodal models, and deep learning models (end-to-end).

Model	Modality	PR-AUC	ROC-AUC	Balanced accuracy	F1 Score	Precision	Sensitivity	Specificity
Logistic Regression	CXR Embeddings	0.8334 [0.8046, 0.8590]	0.7369 [0.7080, 0.7648]	0.6718 [0.6437, 0.6984]	0.7338 [0.7090, 0.7571]	0.7894 [0.7589, 0.8173]	0.6857 [0.6555, 0.7170]	0.6580 [0.6113, 0.7016]
Random Forest	CXR Embeddings	0.7986 [0.7656, 0.8303]	0.7097 [0.6802, 0.7386]	0.6683 [0.6409, 0.6925]	0.7200 [0.6933, 0.7433]	0.7924 [0.7611, 0.8218]	0.6600 [0.6282, 0.6910]	0.6766 [0.6329, 0.7192]
SVM	CXR Embeddings	0.8263 [0.7965, 0.8542]	0.7433 [0.7139, 0.7706]	0.7091 [0.6829, 0.7344]	0.7503 [0.7259, 0.7730]	0.8269 [0.7976, 0.8544]	0.6870 [0.6537, 0.7186]	0.7312 [0.6896, 0.7724]
XGBoost	CXR Embeddings	0.8135 [0.7835, 0.8430]	0.7219 [0.6918, 0.7515]	0.6775 [0.6502, 0.7047]	0.7249 [0.7003, 0.7495]	0.8013 [0.7713, 0.8309]	0.6621 [0.6282, 0.6942]	0.6929 [0.6502, 0.7360]
Logistic Regression	Patient-Level Features	0.8657 [0.8433, 0.8875]	0.7883 [0.7625, 0.8142]	0.7392 [0.7131, 0.7644]	0.8111 [0.7899, 0.8327]	0.8234 [0.7975, 0.8499]	0.7993 [0.7722, 0.8270]	0.6790 [0.6370, 0.7209]
Random Forest	Patient-Level Features	0.9264 [0.9109, 0.9408]	0.8713 [0.8521, 0.8902]	0.8038 [0.7812, 0.8265]	0.8388 [0.8188, 0.8579]	0.8884 [0.8668, 0.9108]	0.7946 [0.7648, 0.8210]	0.8131 [0.7770, 0.8481]
SVM	Patient-Level Features	0.8875 [0.8646, 0.9082]	0.8208 [0.7967, 0.8458]	0.7689 [0.7444, 0.7937]	0.8436 [0.8243, 0.8619]	0.8354 [0.8101, 0.8594]	0.8521 [0.8270, 0.8764]	0.6858 [0.6403, 0.7269]
XGBoost	Patient-Level Features	0.9365 [0.9225, 0.9487]	0.8863 [0.8686, 0.9031]	0.8215 [0.7996, 0.8427]	0.8584 [0.8393, 0.8769]	0.8945 [0.8725, 0.9160]	0.8252 [0.7969, 0.8512]	0.8177 [0.7809, 0.8522]
Logistic Regression	CXR Embeddings and Patient-Level Features	0.8812 [0.8607, 0.9004]	0.7964 [0.7716, 0.8217]	0.7328 [0.7076, 0.7578]	0.7454 [0.7210, 0.7704]	0.8663 [0.8374, 0.8918]	0.6544 [0.6224, 0.6865]	0.8112 [0.7722, 0.8465]
Random Forest	CXR Embeddings and Patient-Level Features	0.8714 [0.8436, 0.8983]	0.8215 [0.7955, 0.8488]	0.7769 [0.7512, 0.8011]	0.8348 [0.8143, 0.8547]	0.8526 [0.8285, 0.8776]	0.8179 [0.7916, 0.8445]	0.7359 [0.6961, 0.7767]
SVM	CXR Embeddings and Patient-Level Features	0.8811 [0.8572, 0.9042]	0.8123 [0.7877, 0.8376]	0.7575 [0.7328, 0.7815]	0.8123 [0.7918, 0.8333]	0.8456 [0.8197, 0.8717]	0.7818 [0.7551, 0.8114]	0.7332 [0.6909, 0.7731]
XGBoost	CXR Embeddings and Patient-Level Features	0.9258 [0.9091, 0.9407]	0.8718 [0.8521, 0.8918]	0.8137 [0.7916, 0.8358]	0.8316 [0.8102, 0.8515]	0.9128 [0.8919, 0.9322]	0.7639 [0.7340, 0.7941]	0.8636 [0.8298, 0.8941]
Multimodal MLP	CXR Embeddings and Patient-Level Features	0.8626 [0.8375, 0.8846]	0.7977 [0.7729, 0.8220]	0.7470 [0.7237, 0.7694]	0.7483 [0.7248, 0.7730]	0.8877 [0.8613, 0.9126]	0.6469 [0.6150, 0.6797]	0.8471 [0.8116, 0.8800]
DEiT-B/16	CXR Images	0.8185 [0.7880, 0.8477]	0.7359 [0.7073, 0.7660]	0.6699 [0.6414, 0.6981]	0.7254 [0.6990, 0.7512]	0.7921 [0.7605, 0.8214]	0.6693 [0.6368, 0.7027]	0.6704 [0.6256, 0.7133]
ViT-B/16	CXR Images	0.8096 [0.7776, 0.8405]	0.7353 [0.7054, 0.7656]	0.6703 [0.6448, 0.6975]	0.7224 [0.6961, 0.7476]	0.7946 [0.7637, 0.8223]	0.6624 [0.6308, 0.6940]	0.6783 [0.6357, 0.7188]
DenseNet-121	CXR Images	0.8073 [0.7766, 0.8352]	0.7151 [0.6855, 0.7448]	0.6535 [0.6260, 0.6806]	0.7248 [0.7008, 0.7502]	0.7729 [0.7430, 0.8019]	0.6826 [0.6504, 0.7146]	0.6244 [0.5782, 0.6689]
ResNet-50	CXR Images	0.8013 [0.7688, 0.8332]	0.7178 [0.6893, 0.7461]	0.6563 [0.6297, 0.6836]	0.7453 [0.7210, 0.7691]	0.7672 [0.7397, 0.7957]	0.7249 [0.6926, 0.7568]	0.5877 [0.5416, 0.6341]

Values in brackets indicate 95% bootstrapped confidence intervals. Classical ML and multimodal models rely on engineered features and multimodal fusion, whereas deep learning models learn representations directly from images (ViT/CNN-based).

In contrast, models trained on patient-level variables achieved substantially stronger performance. XGBoost obtained the highest PR-AUC and ROC-AUC among all classical machine learning models in the patient-level-only setting, with Random Forest and SVM also demonstrating competitive results. These findings highlight the dominant predictive contribution of structured clinical, demographic, and socioeconomic variables.

Adding CXR embeddings to patient-level features resulted in only marginal changes in performance. In several cases, including XGBoost, the patient-level-only configuration even slightly outperformed the multimodal setting, suggesting that imaging features did not consistently contribute additional discriminative value. Multimodal models such as Random Forest and SVM maintained stable but non-improving performance, while the Multimodal MLP achieved comparable results without surpassing classical machine learning approaches, indicating that learned feature fusion does not provide a clear advantage in this dataset.

Among the end-to-end deep learning models trained directly on CXR images (DEiT-B/16, ViT-B/16, DenseNet-121, and ResNet-50), DEiT-B/16 demonstrated relatively more stable performance across metrics. However, as a group, these deep learning models achieved comparable but generally lower performance than patient-level feature-based models. While they provided moderate discrimination, their PR-AUC and ROC-AUC remained below those of the best-performing classical machine learning approaches, particularly those using patient-level features.

This indicates that representation learning from CXR images alone does not capture sufficient predictive signal for DR-TB classification in this cohort. In aggregate, these findings show that routinely collected patient-level variables carry the strongest predictive signal for DR-TB classification, suggesting that clinical history and related covariates are more discriminative for resistance than CXR appearance alone, while imaging-based features, whether engineered or learned via deep learning, provide limited additional gain in this dataset. The consistent pattern across both PR-AUC and ROC-AUC further supports this conclusion under class-imbalanced conditions.

Pairwise DeLong tests further confirmed that XGBoost significantly outperformed all comparator models, including Logistic Regression, Random Forest, SVM, and the multimodal MLP, based on ROC-AUC. This advantage was consistent across all three experimental settings (baseline, without the country variable, and excluding countries containing only DR-TB or DS-TB cases), with minimal variation in both PR-AUC and ROC-AUC for XGBoost, indicating robust performance across data configurations. These findings suggest that XGBoost may represent a reliable approach for supporting clinical decision-making in DR-TB risk assessment. Results are presented in Table 6.

**Table 6:** Performance of multimodal classifiers.

**(A)**			
Model pair	ROC-AUC (XGBoost)	ROC-AUC (comparator)	p-value
XGBoost vs. Logistic Regression	0.8717	0.7957	$3.5\times 10^{-13}$
XGBoost vs. Random Forest	0.8717	0.8212	$7.04\times 10^{-9}$
XGBoost vs. SVM	0.8717	0.8117	$7.07\times 10^{-11}$
XGBoost vs. Multimodal MLP	0.8717	0.7974	$2.11\times 10^{-12}$

**(B)**			
Model	Baseline	Without country	Without single class countries
Logistic Regression	0.8812/0.7964	0.8812/0.8049	0.8751/0.8038
Random Forest	0.8714/0.8215	0.8838/0.8329	0.8718/0.8279
SVM	0.8811/0.8123	0.8845/0.8152	0.8738/0.8108
XGBoost	0.9258/0.8718	0.9234/0.8759	0.9187/0.8739
Multimodal MLP	0.8626/0.7977	0.8495/0.7861	0.8519/0.7917

**(A)** Pairwise DeLong tests comparing XGBoost with other models based on ROC-AUC. **(B)** PR-AUC and ROC-AUC values for each classifier under three experimental settings. Values are reported as PR-AUC/ROC-AUC.

**Figure 4:**
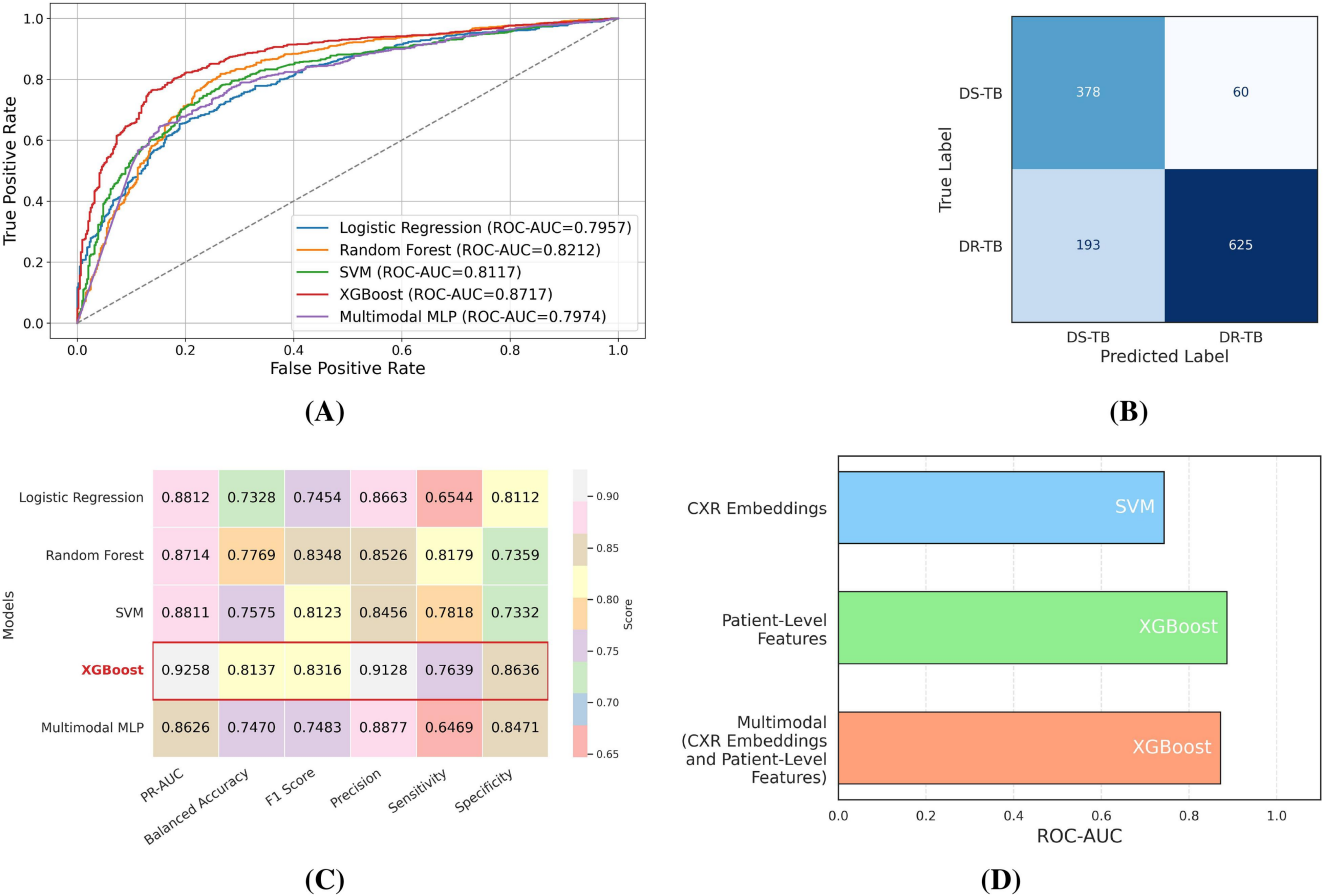
Predictive performance of models using imaging-only, patient-level-only, and multimodal feature sets. **(A)** ROC curves (with AUC values) for XGBoost using combined features. **(B)** Confusion matrix of the best-performing multimodal classifier. **(C)** Heatmap of performance metrics under class imbalance for the leading multimodal model. **(D)** Comparison of best-performing models across modalities based on ROC-AUC.

Figure 4 illustrates these findings, including ROC curves for XGBoost across combined features, the confusion matrix of the top multimodal classifier, a heatmap summarizing multiple performance metrics for the highest-performing multimodal model under imbalanced class conditions, and the best-performing model per modality based on ROC-AUC.

### 4.4 Positioning relative to previous DR-TB and DS-TB classification studies

To contextualize the XGBoost findings, the model was compared with previous DR-TB and DS-TB classification studies, considering dataset size, feature composition, algorithm type, outcomes, and interpretability. As summarized in Table 7, many earlier studies relied on small, chest x-ray-focused datasets with limited clinical information and minimal transparency in model decision-making.

**Table 7:** Comparative overview of DR-TB and DS-TB classification studies using Chest x-ray images.

Study	Images	Features used	Model	Performance	Interpretability
Kovalev et al. (53)	169	Radiological/texture features	SVM	Accuracy = 61.7%	No
Jaeger et al. (54)	135	Radiological/texture features	ANN	Accuracy = 60%, AUC = 66%	No
Tulo et al. (55)	100	Mediastinal, thoracic indices	SVM	F1 Score > 93%	No
Yang et al. (17)	2,237	23 radiological, 2 demographics	SVM	Accuracy = 75%, AUC = 83%	No
Yang et al. (18)	2,622	25 radiological, 7 demographic features	Random Forest	Accuracy = 72.34%, AUC = 78.42%	No
Present Work	6,279	Multimodal features (26 radiological, 3 clinical, 6 demographic/socioeconomic, 1 behavioral, and 378 embeddings)	XGBoost	PR-AUC = 92.58%, ROC-AUC = 87.18%	Yes

By leveraging a large cohort and comprehensive multimodal data, our study achieves superior predictive separation and interpretable outputs. SHAP analysis quantified individual feature contributions, providing insights that build clinician confidence and support integration of model predictions into decision-support workflows. Collectively, these results demonstrate that ensemble-based multimodal modeling offers a scalable, decision-support–ready framework for early DR-TB risk stratification.

### 4.5 Feature importance and model interpretability using SHAP

Interpreting model outputs is critical for transparency and to assess clinical relevance in multimodal DR-TB classification. SHAP analysis was performed on the best-performing XGBoost model to measure global feature importance, evaluate the effects of CXR embeddings and patient-level variables, investigate interactions, and produce sample-specific explanations. This method offers a holistic perspective on model decisions, enhancing clinician confidence and facilitating practical application in patient care.

#### 4.5.1 Global feature importance

**Figure 5:**
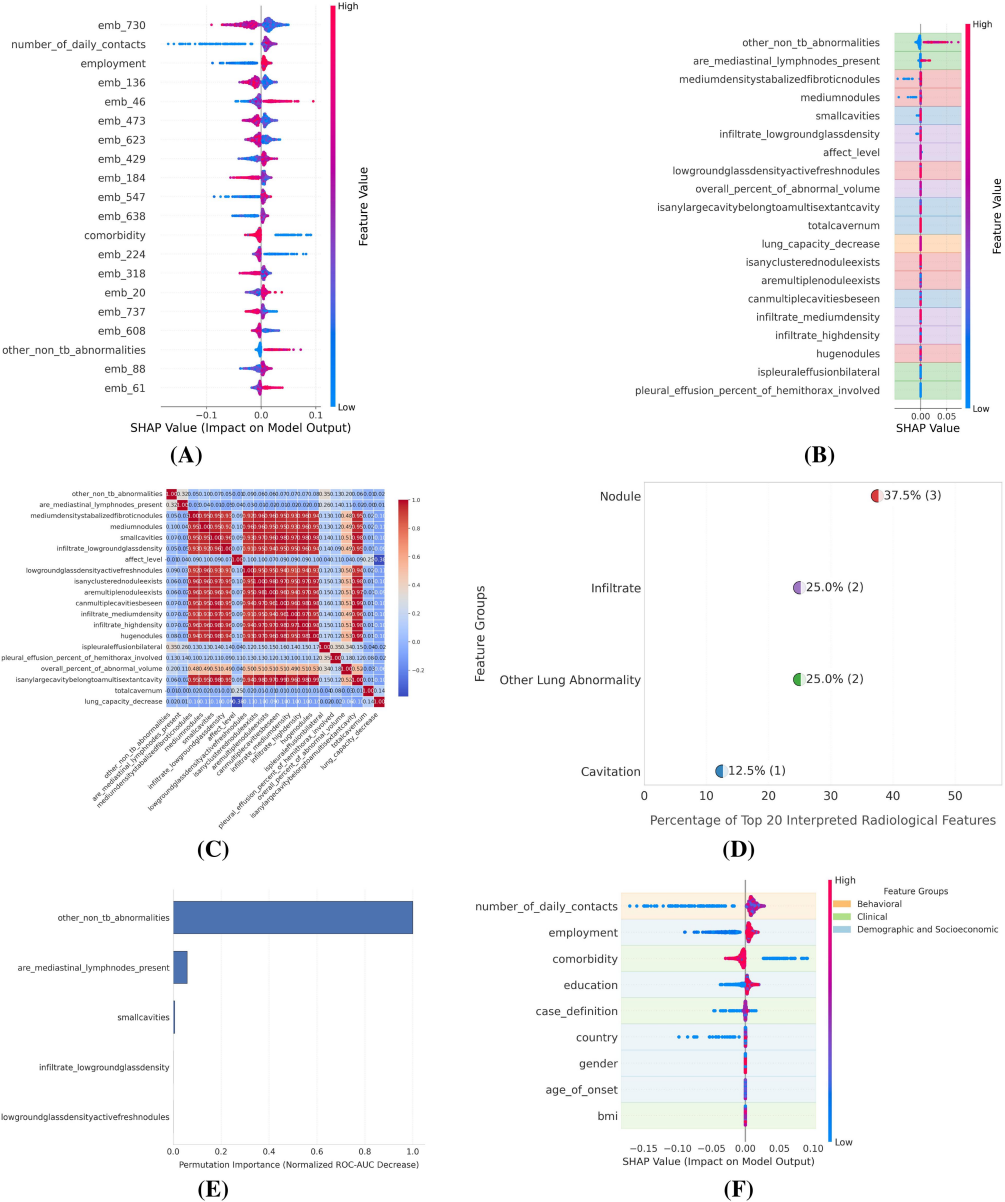
Feature importance and interpretability of the multimodal DR-TB model using SHAP and permutation importance. **(A)** Global SHAP beeswarm plot of the top 20 most influential features across modalities and their contribution to predictions. **(B)** Top radiological features ranked by SHAP importance, highlighting key imaging patterns linked to model output. **(C)** Correlation heatmap of top radiological features, illustrating relationships and redundancy among imaging variables. **(D)** Distribution of radiological feature groups among top predictors, showing dominance of key radiological categories in decision-making. **(E)** Permutation-based feature importance, validating consistency with SHAP attributions. **(F)** SHAP beeswarm plot of clinical, behavioral, and demographic features, showing their contribution to prediction variability.

Figure 5A presents a beeswarm plot of the top 20 features across all patients, while Table 8A summarises their mean SHAP values. The most influential feature overall was a CXR-derived embedding (emb_730), followed by key patient-level variables such as the number of daily contacts, employment status, and comorbidity. In general, CXR embeddings dominated the top-ranked features, accounting for the majority of variables with moderate but consistent contributions to the model. Patient-level features remained important, particularly number of daily contacts and employment, though their overall contributions were more selective. Interpreted radiological features, such as other non-TB abnormalities, appeared among the top predictors but had comparatively smaller effects. In the beeswarm plot, each point represents an individual patient. The x-axis indicates the SHAP value (i.e., the contribution to the predicted probability of DR-TB), and the y-axis lists the features. Point colour reflects the feature value (red = high, blue = low). Higher feature values generally correspond to positive SHAP values, increasing the predicted probability, while lower values tend to decrease it. Overall, the model relies substantially on embedding-derived representations, with patient-level variables providing complementary explanatory signal.

**Table 8:** Feature importance analysis for the multimodal DR-TB prediction model.

Feature	SHAP value	Feature type
**(A)**		
emb_730	0.0202	CXR Embedding
number_of_daily_contacts	0.0158	Patient-Level
employment	0.0114	Patient-Level
emb_136	0.0114	CXR Embedding
emb_46	0.0110	CXR Embedding
emb_473	0.0097	CXR Embedding
emb_623	0.0088	CXR Embedding
emb_429	0.0087	CXR Embedding
emb_184	0.0086	CXR Embedding
emb_547	0.0080	CXR Embedding
emb_638	0.0073	CXR Embedding
comorbidity	0.0073	Patient-Level
emb_224	0.0072	CXR Embedding
emb_318	0.0071	CXR Embedding
emb_20	0.0068	CXR Embedding
emb_737	0.0062	CXR Embedding
emb_608	0.0061	CXR Embedding
other_non_tb_abnormalities	0.0060	Patient-Level
emb_88	0.0059	CXR Embedding
emb_61	0.0055	CXR Embedding
**(B)**		
Feature	SHAP value	Feature type
number_of_daily_contacts	0.0158	Behavioral
employment	0.0114	Socioeconomic
comorbidity	0.0073	Clinical
education	0.0051	Socioeconomic
case_definition	0.0012	Clinical
country	<0.001	Socioeconomic
**(C)**		
Feature	SHAP value	Feature type
other_non_tb_abnormalities	0.0060	Other Lung Abnormality
are_mediastinal_lymphnodes_present	0.0011	Other Lung Abnormality
mediumdensitystabalizedfibroticnodules	<0.001	Infiltrate
mediumnodules	<0.001	Nodule
smallcavities	<0.001	Cavitation
infiltrate_lowgroundglassdensity	<0.001	Infiltrate
affect_level	<0.001	Other Lung Abnormality
lowgroundglassdensityactivefreshnodules	<0.001	Nodule

Feature	Permutation importance	Feature type
**(D)**		
Feature	Permutation importance	Feature type
other_non_tb_abnormalities	0.0213	Other Lung Abnormality
are_mediastinal_lymphnodes_present	0.0012	Other Lung Abnormality
smallcavities	<0.001	Cavitation

Feature	SHAP sensitivity	Prediction sensitivity
**(E)**		
Feature	SHAP sensitivity	Prediction sensitivity
employment	0.0037	0.0069
comorbidity	0.0023	0.0019
number_of_daily_contacts	0.0019	0.0013
mediumdensitystabalizedfibroticnodules	0.0003	0.0003

**(A)** Top 20 global features across all modalities ranked by mean absolute SHAP values. **(B)** Patient-level features grouped by domain. **(C)** Interpreted radiological features grouped by finding category and ranked by SHAP importance. **(D)** Radiological features ranked by permutation importance scores. **(E)** Sensitivity analysis showing SHAP-based and prediction-level feature sensitivity under controlled perturbations.

#### 4.5.2 Radiological feature contributions and group patterns

Analyzing interpreted radiological features separately, both SHAP and permutation importance consistently identified other non-tuberculosis abnormalities as the dominant predictor, clearly outperforming all other features and demonstrating stable agreement across methods, as shown in Figures 5B,E and Table 8. Presence of mediastinal lymphadenopathy ranked second in both analyses, indicating a consistent but substantially weaker contribution to model predictions. All remaining radiological features, including stabilized fibrotic nodules with medium density, pulmonary nodules, small cavities, infiltrates with low ground-glass attenuation, lesion extent level, and active low-density ground-glass nodular opacities, contributed only marginal or negligible influence across both attribution methods, reflecting limited incremental value beyond the dominant and secondary predictors. Taken together, both SHAP and permutation importance analyses indicate a strongly skewed feature importance distribution, dominated by non-tuberculosis abnormalities, followed by mediastinal lymph node involvement, with all other radiological features providing only residual signal.

Grouping of features showed that nodules were the most prevalent radiological feature among the top 20 interpreted predictors. This was followed by infiltrates and other lung abnormalities, which appeared with comparable frequency, while cavitation was the least frequent feature. Markedly, features associated with lung inflation or collapse were not observed among the top predictors, as illustrated in Figure 5C. Correlation analysis revealed strong clustering between cavitation and nodular features, with moderate associations involving infiltrative and ground-glass abnormalities, as shown in Figure 5D. In contrast, pleural effusion and global volume-based measures exhibited weak correlations with most texture-level features, indicating relative independence. These structured relationships are consistent with established radiologic progression in drug-resistant tuberculosis and support the model’s interpretability for assessing localized structural lung damage.

#### 4.5.3 Clinical, behavioral, and demographic drivers

Patient-level characteristics collectively influenced model predictions, with behavioral variables demonstrating the strongest overall impact. The number of daily contacts was the most influential feature, followed by employment status, highlighting the importance of exposure intensity and occupational context in shaping drug-resistant tuberculosis risk. Clinical comorbidity contributed a moderate level of influence, while education had a comparatively smaller effect. In contrast, case definition showed only minimal contribution, and country of origin had a negligible impact, as reflected in the Mean Absolute SHAP values and summarized in Table 8C, with supporting visual patterns presented in Figure 5F. In this context, the results indicate that model predictions are primarily driven by behavioral exposure factors, with clinical characteristics playing a secondary role and demographic variables offering limited additional explanatory value.

#### 4.5.4 Feature interactions

**Figure 6:**
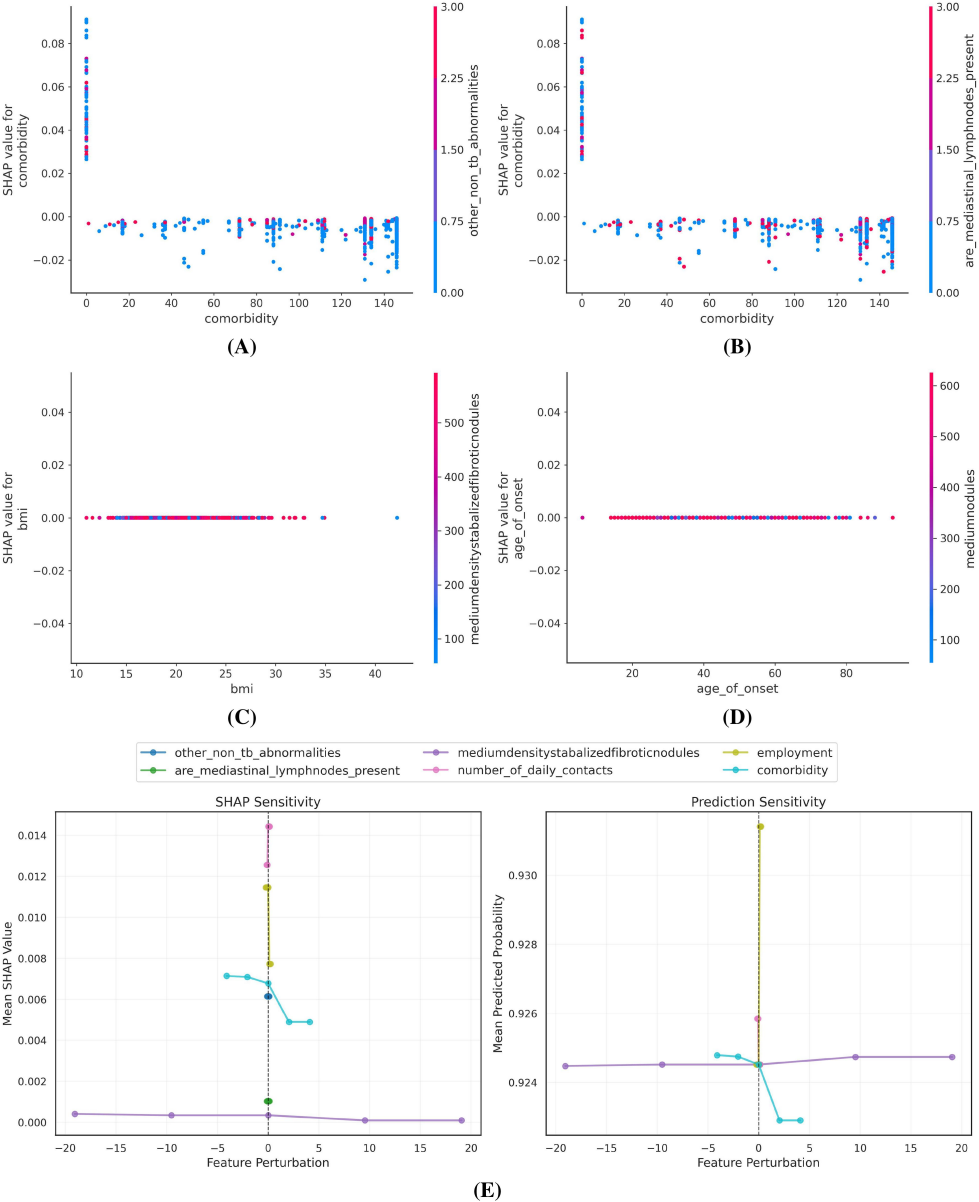
Model interpretability and robustness analysis. SHAP dependence plots **(A–D)** showing context-dependent variation in the influence of clinical variables across radiological feature space (not formal statistical interaction effects). **(E)** Sensitivity analysis of model explanations and predictions under feature perturbations, assessing stability of SHAP attributions and output predictions.

Figures 6A–E illustrates context-dependent feature interactions and sensitivity behavior. SHAP dependence plots (A,B) show that comorbidity contributes positively to predicted DR-TB risk primarily at low values, with higher SHAP effects in the presence of other non-TB abnormalities or mediastinal lymph node involvement, while contributions attenuate at higher comorbidity levels. BMI and age at onset demonstrate largely flat SHAP profiles with minimal interaction effects across nodular burden (C,D). Sensitivity analysis under controlled feature perturbations (E) and quantifies feature influence at both the prediction and explanation levels (56). Employment status emerges as the dominant driver of prediction-level variation, while comorbidity and number of daily contacts also contribute meaningfully. At the attribution level, SHAP-based sensitivity indicates that employment and comorbidity have the strongest influence, followed by number of daily contacts and radiological features. Detailed sensitivity values are provided in Table 8E. Together, the findings show that different features govern explanation-level attribution and prediction-level sensitivity, highlighting stable and interpretable model behavior.

#### 4.5.5 Local explanations (sample-level SHAP)

**Figure 7:**
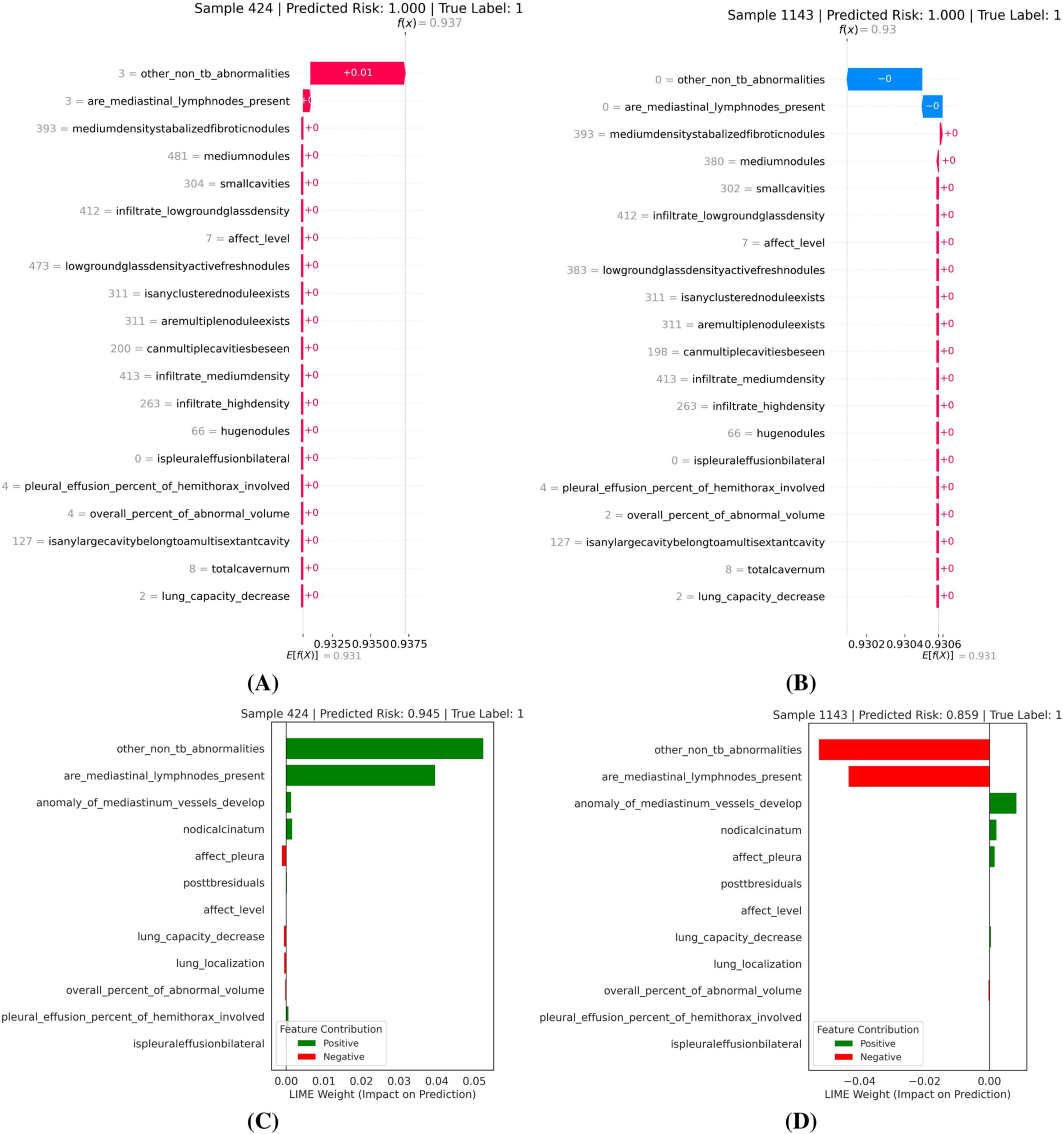
Waterfall plots of SHAP values for two high-risk patients **(A,B)**, showing the additive contribution of the top 20 interpreted radiological features to the predicted DR-TB risk. Positive bars increase, and negative bars decrease the predicted probability, illustrating the relative influence of each feature on patient-specific risk. Corresponding local explanations using LIME are shown in **(C,D)**, highlighting feature contributions for the same patients (Sample 424 shown as example with predicted risk 0.945), and enabling comparison between SHAP and LIME-based interpretability methods.

At the level of individual predictions, Figures 7A–D presents SHAP and LIME feature attributions for two high-risk patients using the same samples. SHAP waterfall plots (A,B) decompose each prediction relative to the baseline expectation (E[f(x)]≈0.931), yielding final predicted probabilities of f(x)=0.937 and f(x)=0.930. Consistent with the small magnitude of the bars shown across both methods, the displayed radiologic features produce only incremental shifts around the baseline, with modest positive contributions from non-TB abnormalities and selected cavitary or infiltrative features, while nodular patterns, pleural effusion measures, and global volume indicators remain close to zero. Variations in sign and magnitude between the two patients indicate context-dependent local effects rather than a fixed or global feature ranking.

Corresponding LIME explanations (C,D) provide local linear approximations of feature influence for the same predictions. For Sample 424, non-TB abnormalities and mediastinal lymph node involvement exert positive contributions, whereas for Sample 1,143 these features contribute negatively, illustrating local reversals in feature impact depending on the surrounding feature context. Other radiologic features show smaller LIME weights, indicating limited marginal influence at the individual level. Together, SHAP and LIME consistently demonstrate that radiologic features support interpretable patient-level explanations but account for only a portion of the final prediction, which also incorporates clinical, demographic, behavioral, socioeconomic variables, and learned CXR embeddings not shown here.

### 4.6 Clinical consistency of SHAP interpretations

Model explanations were evaluated by two independent clinicians with expertise in tuberculosis and infectious diseases across 10 representative DR-TB and DS-TB cases, following the procedure described in Section 3.7. Findings indicate that feature contributions were broadly consistent with established epidemiological, radiological, and clinical patterns of tuberculosis as reported in Supplementary Appendix S1. Key determinants, including clustered nodular disease, cavitary changes, low BMI, anemia as a comorbidity, exposure history, and socioeconomic vulnerability, were identified as contributors to predicted DR-TB risk. Radiological features such as mediastinal lymph node involvement and non-tuberculous abnormalities were generally interpreted as non-specific indicators of overall disease burden rather than TB-defining features. Taken together, these findings support the interpretability and clinical validity of the framework.

## 5 Discussion

Drug-resistant tuberculosis remains a major global health challenge, highlighting the importance of distinguishing DR-TB from drug-sensitive TB to guide treatment and population-based interventions. Using data from 6,279 subjects in the TB Portals program, we developed an interpretable multimodal machine learning model that integrates chest x-ray embeddings from a Data-Efficient Image Transformer with patient-level features, including clinical, interpreted radiological, demographic, socioeconomic, and behavioral variables.

In the multimodal configuration, XGBoost achieved the highest PR-AUC and ROC-AUC, outperforming other algorithms. This superior performance is consistent with XGBoost’s gradient boosting framework, which iteratively combines weak learners and effectively handles heterogeneous, high-dimensional data, enabling the capture of nonlinear interactions between imaging and patient-level features. By contrast, models such as Random Forest and logistic regression may be less effective at leveraging subtle cross-feature dependencies in multimodal inputs. In particular, Random Forest attained the highest PR-AUC and ROC-AUC when using patient-level features alone, reinforcing that optimal model choice remains dependent on the feature set and that incorporating imaging features does not necessarily yield additional performance gains (42, 43).

Notably, the comparative analysis demonstrated that patient-level features alone achieved the highest overall discrimination, while multimodal integration and deep learning approaches, including the Multimodal MLP, did not yield substantial performance gains. This suggests that clinical and demographic variables capture most of the predictive signal for DR-TB in this cohort, and that imaging features, whether represented as embeddings or learned end-to-end, provide only limited incremental value.

The comparable performance of deep learning and classical machine learning models on imaging inputs further indicates that increased model complexity does not necessarily translate into improved predictive performance, particularly in settings with limited sample size or feature redundancy. This may be explained by the suitability of tree-based models such as XGBoost and Random Forest for structured, heterogeneous tabular data, where they can effectively capture non-linear interactions with relatively small sample sizes, whereas deep learning models typically require larger datasets and are less efficient when applied to tabular features.

However, it is important to note that these analyses were performed within an internal validation framework. While the cohort includes data from multiple countries, the distribution is uneven across regions, which may limit full cross-population generalizability. More comprehensive external validation strategies, such as leave-one-country-out evaluation, represent an important direction for future work to further assess robustness across geographically distinct populations.

To our knowledge, this is the first multimodal machine learning framework providing interpretable insights at both modality-level and global feature attribution levels for integrating CXR embeddings with patient-level features for differential identification of DR-TB. Compared with prior studies relying solely on chest x-ray images (53–55) or limited clinical, radiological, and demographic data (17, 18), the proposed multimodal framework achieves superior discrimination between DR-TB and DS-TB while offering explainable insights into the most influential predictors. This combination of predictive accuracy and interpretability underscores the clinical utility of multimodal models for guiding targeted interventions and informing public health strategies in high-burden, resource-limited contexts (8).

To further interpret these findings, Shapley Additive Explanations revealed that the model was primarily driven by CXR-derived embedding features, followed by behavioral and clinical variables. Among patient-level features, number of daily contacts and employment status were the strongest contributors, highlighting the importance of exposure and socioeconomic factors. Comorbidity contributed moderately, while education and case definition showed smaller effects.

Interpreted radiological features such as other non-tuberculosis abnormalities and mediastinal lymph node involvement provided additional predictive signal, whereas medium-density stabalized fibrotic nodules, medium nodules, and small cavities contributed slightly and most other radiological findings contributed marginally (57). Demographic variables, including country of origin, had negligible impact on predictions.

The results align closely with established risk factors for DR-TB reported in the literature (18, 58–61), supporting the role of exposure-related factors, host status, and structural lung abnormalities in disease prediction. Collectively, the model relied predominantly on learned imaging representations, complemented by key behavioral and clinical risk factors. Importantly, an independent assessment by two clinicians specialising in tuberculosis and infectious diseases confirmed the plausibility and alignment of SHAP-based explanations with domain knowledge, reinforcing their interpretability in practice.

Feature interactions demonstrated context-dependent effects between clinical, behavioral, and radiological variables. Comorbidity showed a non-linear relationship with predicted DR-TB risk, contributing more strongly at lower levels and interacting with other non-tuberculosis abnormalities and mediastinal lymph node involvement, while its effect diminished at higher levels. BMI and age at disease onset exhibited largely flat effects with minimal interaction patterns across radiological feature space, indicating limited context-dependent influence (62).

Sensitivity analysis revealed a consistent hierarchy of feature influence across both prediction and attribution levels. Employment status was the primary driver of prediction variability, followed by comorbidity and the number of daily contacts, while radiological features showed minimal impact across both perspectives (63). These patterns indicate a largely aligned structure between prediction drivers and explanatory features, with socio-demographic and clinical variables contributing more prominently than imaging-derived features. This analysis further highlights model robustness by distinguishing between features driving prediction changes and those contributing to model attribution, supporting interpretability of the model.

The model supports timely identification of drug-resistant tuberculosis, helping prioritize testing and treatment. Predictions can be generated automatically upon registration or chest x-ray acquisition, flagging cases for expedited drug susceptibility testing, closer monitoring, or timely therapy. In addition, these predictions can guide treatment planning by highlighting patients who may require early initiation of second-line therapies, more intensive monitoring, or tailored drug regimens based on their individualized risk profiles. This approach complements, but does not replace, confirmatory testing, supporting efficient resource allocation and care.

At the population level, aggregated model predictions can support targeted screening, contact tracing, and efficient allocation of limited diagnostic and public health resources. Model outputs can be used to prioritize individuals with higher predicted risk for further diagnostic evaluation and follow-up, thereby supporting more efficient use of available healthcare resources in high-burden settings. While local SHAP and LIME explanations demonstrate consistent patient-level interpretability, these insights primarily support decision-making at the individual level rather than explicit identification of population-level clusters (52).

The interpretable, multimodal framework could be integrated with electronic health records and digital triage platforms to support early identification of high-risk DR-TB patients. In low-resource settings, where access to rapid diagnostics is limited, automated risk prediction could help prioritize patients for confirmatory testing, allocate scarce treatment resources efficiently, and guide targeted community screening campaigns.

While the model demonstrates strong accuracy and potential utility, several limitations warrant consideration. Its retrospective design and evaluation within a single cohort may limit generalizability across diverse populations, healthcare systems, and epidemiological contexts. Feature attributions remain associative rather than causal. In addition, real-world clinical deployment may involve noisy or incomplete data, including missing patient-level variables and variability in chest x-ray quality.

Future work should validate the model across broader populations, refine decision thresholds through targeted analyses of misclassified high-risk patients, systematically evaluate robustness under missing and noisy data conditions, assess integration into clinical workflows, and examine feasibility and operational impact in low-resource settings. Evaluating patient impact and implementation feasibility will be essential for translating these findings into improvements in clinical care and public health. Incorporating strategies such as data imputation, uncertainty estimation, and robustness-aware training may further enhance reliability in real-world settings. Future research could also explore hybrid modelling approaches that integrate data-driven methods with epidemiological or mechanistic priors to better capture disease processes, although their applicability to multimodal clinical prediction remains to be established.

## 6 Conclusion

This study demonstrates that an interpretable machine learning approach can effectively classify tuberculosis cases as drug-resistant or drug sensitive. Patient-level features, particularly the number of daily contacts and employment status, were the strongest predictors of DR-TB, with comorbidity contributing moderately. The strong contribution of the number of daily contacts likely reflects increased social mixing and exposure intensity, both of which are key determinants of transmission risk in high-burden settings. Employment status may be indicative of underlying socioeconomic conditions, exposure patterns, and access to care, all of which influence both transmission risk and healthcare utilisation. Interpreted radiological variables, including other non-tuberculosis abnormalities and mediastinal lymph node involvement, provided additional discriminative information, while medium nodules and small cavities contributed slightly. These findings highlight the relevance of structural lung pathology in DR-TB risk stratification. Incorporating CXR embeddings extracted using a Data-Efficient Image Transformer added complementary structural information by capturing subtle imaging patterns not readily apparent through routine interpretation; however, their incremental contribution to predictive performance remained limited compared to patient-level features. Context-specific interactions further indicated that model predictions are shaped by non-linear relationships between clinical, behavioral, and radiological variables, supporting a more nuanced representation of DR-TB risk. Crucially, an independent evaluation conducted by two clinicians specialising in tuberculosis and infectious diseases confirmed that the SHAP-based explanations were plausible and consistent with established domain knowledge. Aggregated predictions can support targeted screening, contact tracing, and resource allocation, enabling both individualized patient management and more efficient public health interventions in TB-endemic settings. Future work should focus on external validation across diverse high-burden populations, refinement of risk thresholds through analysis of misclassified cases, and evaluation of integration into clinical workflows to translate these findings into improved patient outcomes and more effective TB control strategies.

## Data Availability

The datasets analyzed for this study are available from the TB Portals Program upon reasonable request, subject to approval. Code supporting the analyses, including the novel multimodal integration pipeline and SHAP interpretability framework, is publicly hosted on GitHub: https://github.com/jjmnyambo/DRTB-Multimodal-Interpretable-ML.
