# Supplementary material for: Interpretable multimodal machine learning for diagnosis of drug-resistant tuberculosis
Source: Front Digit Health. 2026 Jul 30;8:1828522. doi: 10.3389/fdgth.2026.1828522 (PMC13474479; doi:10.3389/fdgth.2026.1828522)
Supplement: Supplementary file 1 [file Supplementaryfile1.pdf]

Clinical Review of SHAP-Based Interpretations of a Multimodal Drug-Resistant Tuberculosis Machine Learning Model

Purpose

This document presents 10 representative patient-level cases selected for clinical validation of SHAP-based explanations from a multimodal machine learning model distinguishing drug-resistant tuberculosis (DR-TB) from drug-sensitive tuberculosis (DS-TB). Each case integrates interpreted radiological features and non-imaging patient-level variables contributing to model predictions and supports multidisciplinary clinical review.

Variable Definitions

- **Age:** Reported in years at clinical presentation
- **BMI:** Body mass index reported in kg/m^2

Overall Clinical Feedback

Clinical review confirmed that SHAP-identified features align with established epidemiological and clinical determinants of DR-TB. Influential non-imaging factors included anemia, contact exposure, socioeconomic vulnerability, prior TB history (new/relapse/failure), and low BMI. Radiological patterns included clustered nodular disease, mixed ground-glass and fibrotic nodules, infiltrates, and cavitation. Non-tuberculous abnormalities and mediastinal lymph nodes acted as contextual rather than TB-defining features. Overall, SHAP explanations were clinically plausible and consistent with known tuberculosis phenotypes.

Case Summaries

Patient 504 — DR-TB (True Positive)
Prediction: 1.000 | **Ground truth**: DR-TB
Interpreted Radiological Features: Extensive clustered nodular disease with mixed ground-glass (active) and fibrotic (chronic) changes, infiltrates, and small cavitary lesions; no mediastinal lymphadenopathy.
Non-Imaging Patient-Level Variables: Comorbidity (anemia), socioeconomic vulnerability, contact exposure, new case, female, 71 years, BMI 18.7
Interpretation: Advanced mixed active-chronic pulmonary TB with features associated with increased DR-TB risk
Reviewer Note: Advanced TB pattern with significant disease burden; microbiologic confirmation is required.

Patient 424 — DR-TB (True Positive)
Prediction: 1.000 | **Ground truth**: DR-TB
Interpreted Radiological Features: Clustered nodular disease with mixed ground-glass and fibrotic components and infiltrates; contextual non-TB abnormalities
Non-Imaging Patient-Level Variables: Comorbidity (anemia), exposure, socioeconomic vulnerability, relapse, male, 37 years, BMI 18.5
Interpretation: Relapse-associated mixed active--chronic TB with increased DR-TB risk
Reviewer Note: Mixed active–chronic pulmonary TB; microbiological confirmation required.

Patient 1143 — DR-TB (True Positive)
Prediction: 1.000 **| Ground truth:** DR-TB
Interpreted Radiological Features: Extensive nodular disease with mixed ground-glass and fibrotic changes; absence of competing abnormalities
Non-Imaging Patient-Level Variables: Comorbidity (anemia), exposure history, socioeconomic vulnerability, new case, male, 40 years, BMI 15.6
Interpretation: Severe pulmonary TB with systemic vulnerability and increased DR-TB risk
Reviewer Note: Radiology supports advanced TB but not definitive drug resistance; microbiology remains essential
Additional Clinical Note: Prefer malnutrition-associated risk rather than assuming immune compromise unless clinically confirmed.

Patient 144 — DR-TB (True Positive)
Prediction: 0.999 | **Ground truth**: DR-TB
Interpreted Radiological Features: Clustered nodular disease with mixed ground-glass and fibrotic nodules, infiltrates, and mediastinal lymph node involvement; non-TB abnormalities as contextual signals
Non-Imaging Patient-Level Variables: Comorbidity (anemia), socioeconomic vulnerability, exposure, new case, female, 25 years, BMI 16.9
Interpretation: Advanced nodular TB with features suggestive of increased DR-TB risk
Reviewer Note: Extensive disease with systemic vulnerability; high-risk profile for severe TB

Patient 147 — DR-TB (True Positive)
Prediction: 0.999 | **Ground truth**: DR-TB
Interpreted Radiological Features: Clustered nodular disease with mixed active and fibrotic changes and infiltrates; no competing abnormalities
Non-Imaging Patient-Level Variables: Comorbidity (anemia), exposure history, socioeconomic vulnerability, treatment failure, male, 63 years, BMI 17.7

Interpretation: Advanced pulmonary TB with strong clinical risk factors for drug resistance
Reviewer Note: Treatment failure significantly increases probability of DR-TB

Patient 613 — DR-TB (True Positive)
Prediction: 0.999 | **Ground truth**: DR-TB
Interpreted Radiological Features: Non-specific nodular and infiltrative pattern consistent with a TB imaging profile
Non-Imaging Patient-Level Variables: Comorbidity (anemia), exposure, socioeconomic factors, treatment failure, female, 30 years, BMI 17.5
Interpretation: TB-consistent imaging with high-risk clinical profile suggestive of DR-TB
Reviewer Note: Clinical context strongly supports elevated DR-TB risk despite non-specific imaging

Patient 966 — DS-TB (True Negative)
Prediction: 0.855 | **Ground truth**: DS-TB
Interpreted Radiological Features: Mild clustered nodules and infiltrates without cavitation or advanced patterns
Non-Imaging Patient-Level Variables: Exposure, socioeconomic factors, new case, male, 39 years, BMI 20.6, comorbidity (anemia)
Interpretation: Non-advanced pulmonary TB consistent with DS-TB
Reviewer Note: Clinical and imaging findings support drug-sensitive disease

Patient 1211 — DS-TB (True Negative)
Prediction: 0.539 | **Ground truth**: DS-TB
Interpreted Radiological Features: Limited nodular disease with mild inflammatory and fibrotic changes; no cavitation
Non-Imaging Patient-Level Variables: Exposure, socioeconomic factors, new case, male, 57 years, BMI 26.5, comorbidity (anemia)
Interpretation: Mild TB with limited severity and low likelihood of drug resistance
Reviewer Note: Preserved nutritional status supports lower disease burden

Patient 1063 — DS-TB (True Negative)
Prediction: 0.441 | **Ground truth**: DS-TB
Interpreted Radiological Features: Mild nodular and infiltrative changes without cavitation or advanced destructive patterns
Non-Imaging Patient-Level Variables: Exposure, socioeconomic factors, relapse case, male, 48 years, BMI 16

Interpretation: TB pattern consistent with DS-TB despite nutritional risk
Reviewer Note: Imaging does not support advanced or drug-resistant disease

Patient 0 — DR-TB (False Positive)
Prediction: 0.954 | **Ground truth**: DS-TB
Interpreted Radiological Features: Extensive nodular disease with mixed ground-glass, fibrotic, and infiltrative changes overlapping DR-TB-like patterns
Non-Imaging Patient-Level Variables: Exposure, socioeconomic factors, new case, male, 23 years, BMI 16, comorbidity (anemia)
Interpretation: Misclassification due to imaging overlap with severe TB patterns
Reviewer Note: Clinical context favors DS-TB; overlap between active and chronic changes explains false-positive prediction

Clinician Statement of Agreement

I have reviewed the SHAP-based case interpretations presented in this document and confirm that the interpreted radiological, clinical, demographic, socioeconomic, and behavioral feature attributions are clinically plausible and consistent with the current understanding of drug-resistant tuberculosis.
